# A Mathematical Dimensional Model for Predicting Bulk Density of Inconel 718 Parts Produced by Selective Laser Melting

**DOI:** 10.3390/ma14030512

**Published:** 2021-01-21

**Authors:** Jorge A. Estrada-Díaz, Alex Elías-Zúñiga, Oscar Martínez-Romero, J. Rodríguez-Salinas, Daniel Olvera-Trejo

**Affiliations:** School of Engineering and Science, Tecnológico de Monterrey, Av. E. Garza Sada 2501 Sur, Monterrey 64849, Mexico; oscar.martinez.tec@gmail.com (O.M.-R.); juanjrdz@tec.mx (J.R.-S.); daniel.olvera.trejo@tec.mx (D.O.-T.)

**Keywords:** selective laser melting, dimensional analysis, manufacturing parameters, Inconel 718, AlSi10Mg, Ti6Al4V

## Abstract

**Featured Application:**

**Mathematical tool for tuning Selective Laser Melting process parameters for achieving highly dense components.**

**Abstract:**

In this work, dimensional analysis is used to develop a general mathematical model to predict bulk density of SLMed components taking volumetric energy density, scanning speed, powder’s thermal conductivity, specific heat capacity, and average grain diameter as independent variables. Strong relation between dependent and independent dimensionless products is observed. Inconel 718 samples were additively manufactured and a particular expression, in the form of a power-law polynomial, for its bulk density, in the working domain of the independent dimensionless product, was obtained. It is found that with longer laser exposure time, and lower scanning speed, better densification is attained. Likewise, volumetric energy density has a positive influence on bulk density. The negative effect of laser power in bulk density is attributed to improper process conditions leading to powder particle sublimation and ejection. A maximum error percentage between experimental and predicted bulk density of 3.7119% is achieved, which corroborates the accuracy of our proposed model. A general expression for determining the scanning speed, with respect to laser power, needed to achieve highly dense components, was derived. The model’s applicability was further validated considering SLMed samples produced by AlSi10Mg and Ti6Al4V alloys. This article elucidates how to tune relevant manufacturing parameters to produce highly dense SLM parts using mathematical expressions derived from Buckingham’s π- theorem.

## 1. Introduction

### Selective Laser Melting

Additive manufacturing (AM) has gained interest in industrial spheres due to its benefits, reduction of waste materials, shortening of manufacturing times, high flexibility, production of complex geometry products, shortening of product development cycle, among others [1]. Wohlers Associates’ “Annual Worldwide Report on 3D Printing and Additive Manufacturing” states that the global revenue generated by AM production and associated services will reach $21 billion by 2020 [2].

Selective laser melting (SLM) is an AM powder bed fusion technology which involves heating, melting, and solidification of a metallic powder by a moving heat source, in the form of a laser, in a layer-by-layer manner [3]. Each scanning process produces a thin cross-sectional layer of the final product. The final component is, therefore, completed in an iterative process of depositing feedstock, laser scanning, melting the feedstock, and undergoing a solidification lapse [4]. Once the repetitive process is completed, loose powders are removed from the building chamber and the produced piece is separated from the substrate plate either manually or by electrical discharge machining, as required [5].

Through SLM, it is possible to produce metallic parts with intricate geometries with high three-dimensional accuracy [6]. The sectors where SLM has found its most significant contributions are in high value-added industries, such as aerospace, automotive, and medicine [5]. For instance, nickel-based super alloy abrasive turbine blade tips, which reduced gas leakage, and thus improved turbine efficiency, were produced [7]. Aluminum-based metal matrix composites that exploit the lightweight, high specific strength, and thermal conductivity of aluminum for aerospace and automotive applications were investigated by Dadbakhsh et al. [8]. A proposal for a potential porous implant or drug delivery system using NiTi (known for its high strength, high corrosion resistance, biocompatibility, and shape memory effect) with hydroxyapatite additions was given by Shishkovskii et al. [9].

Inconel 718 is chosen as the material of study for this research since it is of high relevance in the aerospace and biomedical industries. It is used to produce critical components in aircraft, gas turbines, turbocharger roots, nuclear reactors, liquid fueled rockets, and several corrosive and structural applications involving high operation temperatures [10]. Ni-Cr-based superalloy is chosen for its strength, creep resistance, good weldability, and fatigue life at temperatures up to 700 °C [11]. Aircraft engine components such as critical rotating parts, aerofoils, supporting structures, and pressure vessels (which represent approximately 30% of the aircraft’s total weight) have been produced with Inconel 718 [12]. In718 is a hard-cutting alloy with hardness (Brinell) HB values ranging from 240 to 410 kg/mm^2^, and thus, the SLM of this material represents a potentially relevant application for complex geometries working in highly abrasive conditions. Kapłonek et al. [13] performed studies on the grinding process of In718 rings with high-efficiency MoS_2_ treated grinding wheels with promising positive results.

SLM process parameters must be adjusted such that a single melt line can fuse completely with the neighboring melt trajectories and the preceding layer [5]. Spears et al. discuss additional details in [14]. There, the authors divide the process parameters in four categories: (1) laser and scanning parameters, (2) powder material properties, (3) powder bed properties and recoat parameters, and (4) build environment parameters. Of the fifty parameters listed, only twelve can be directly adjusted for the process [14]. Among these, it may be listed: laser power, spot size, scan velocity, spacing and strategy, deposition system, layer thickness, powder bed temperature, oxygen level, chamber pressure, temperature, and gas flow velocity.

Most publications on SLM focus on studying the influence that process parameters have on a vast variety of physical and structural properties of AM metallic samples. An overview of such studies is provided in Table 1. Post-processing is also relevant in SLM. Waqar et al. [15] found that microstructure and mechanical properties of SLMed SS316L components are influenced by different post-annealing conditions at which the cooling down takes place. Likewise, Gupta et al. [16] found that higher angle layer rotation leads to a reduction in grain size, residual stress, and surface roughness. Moreover, micro-hardness, tensile strength, elongation, and relative density were significantly enhanced. Khanna et al. [17] performed a study on Invar-36, a Ni-Fe-based alloy used in the aerospace industry and known for its low thermal expansion, of the effect of laser power, scan speed, layer thickness, and hatch spacing on the SLSed (Selective Laser Sintering) densification and surface roughness.

In the SLM process, volumetric energy density is defined by the quotient of laser power supply and the product of scanning speed with hatch spacing, layer thickness, laser spot size, or average powder diameter. The effect of volumetric energy density (VED) on melt pool dimensions and geometry, densification, surface roughness, dimensional accuracy, microstructure, hardness, fatigue life and mechanical properties have been studied on a wide variety of materials. A brief overview of these performed studies is presented in Table 2. Even though VED has been thoroughly investigated, authors like Scipioni et al. [28] and Mishurova et al. [29] have suggested that VED is not able to fully describe the SLM physical process and therefore, one should use VED with caution as a design parameter.

Besides the experimental design methodologies, computational algorithms are also used to study the SLM process [41]. Finite element method (FEM) models, in SLM, have been developed to study the deposition process, part distortion, and thermal gradients [42], heat distribution and residual stress [43], temperature profile and melting process [44], melt pool size [45], just to mention a few. Adaptive meshing has been used to determine thermal gradients near laser incidence points [46]. Moser et al. [47] presented a part scale continuum model predicting thermal stresses which incorporate thermal, laser, and mechanical properties for SS316L. Ahmadi et al. [48] developed a computational model which studied the response of the mechanical properties of SLMed SS316L to a series of process parameters. The multi-track, multi-layer, and multi-material SLM process was modeled by the discrete element method [49]. Numerical tools were developed for obtaining reliable processing windows [50] and studying the thermal behavior, and melt pool morphology in the multi-track multi-layer SLM of SS316L [51].

The application of dimensional analysis to selective laser melting has not been studied thoroughly in the research community. Van Elsen et al. presented a possible complete set of dimensionless parameters to describe the process, aiming at facilitating comparison between different research groups works [52]. Cardaropoli et al. used dimensional analysis intending to find out an appropriate definition of a set of non-dimensional groups in order to represent the output parameters for the process [53], proposing a set of 16 independent physical quantities and twelve π-products evaluated in Ti6Al4V. Most recently, Khan et al. [54] used dimensional analysis to model heat source in SLM as a function of laser parameters and properties of alloy powder in SS316L.

In this work, dimensional analysis is used to develop a general mathematical model to predict the bulk density of SLMed components, taking into account relevant process parameters and properties such as volumetric energy density, scanning speed, powder’s thermal conductivity, specific heat capacity, and average diameter. In comparison with previous studies, the reduced number of elements of this independent set allows the full evaluation of the physical model through experimentation. Moreover, high precision for the prediction of bulk density is attained, proving that in fact, dimensional analysis has succeeded at describing the behavior of the densification of SLMed components, and that the chosen set is adequate. Moreover, this proposed model is able to identify a mathematical expression for determining the scanning speed value needed to achieve high part densification, with respect to the laser power supply, and vice versa. This expression is of high practical relevance since it allows the user to tune manufacturing parameters to obtain highly dense SLMed metallic components, leading to a potential significant reduction on material waste and costs associated to experimentation.

Validation of the developed model is primarily addressed by using information from produced Inconel 718 SLMed samples in which bulk density was predicted using, for instance, the laser scanning speed. The validity of the mathematical formulation through different materials is confirmed with adapted experimental data collected from AlSi10Mg and Ti6Al4V.

The purpose of this work is to obtain a general mathematical expression which is able to predict the bulk density of a metallic component manufactured through selective laser melting, as well as to develop a mathematical tool for properly defining the needed scanning speed, with respect to laser power, to attain highly dense pieces.

## 2. Materials and methods

### 2.1. Modelling AM by Dimensional Analysis

#### 2.1.1. Introduction to Dimensional Analysis

Before we start with the derivation of a mathematical model based on Buckingham’s π-theorem to find the relation between process parameters and physical phenomena, we briefly review some definitions and basic foundations of dimensional analysis.

The basis of dimensional analysis is that a physical phenomenon can be described by the following relationship [55]:

(1) $$f\left(Q_{0},Q_{1},\ldots ,Q_{n}\right)=0$$

where $Q_{i},\;i\;=\;0,\;1,\;2,\;\ldots ,\;n$ represents a property in the general thermodynamic sense or a physical quantity. Assuming an interest in some dependent particular physical quantity *Q*_0_, we have that [55]:

(2) $$Q_{0}=f(Q_{1},Q_{2},\ldots ,Q_{n-1})$$

As a whole, $Q_{i},\;i\;=\;1,\;2,\;\ldots ,\;n-1$ is a complete set of independent physical quantities. Defining these factors is the first and most important step in dimensional analysis. Then, a complete, dimensionally independent, subset $Q_{1},\;\ldots {,\;Q}_{k}$ is chosen from the complete independent set $Q_{1},\;\ldots {,\;Q}_{n-1}$. The dimensions of the dependent and remaining independent physical quantities are expressed as a power law of the dimensions of the dimensionally independent subset. Dimensionless products are constructed from these equidimensional products.

Buckingham-π theorem of dimensional analysis transforms Equation (1) into [55]:

(3) $$f\left(\pi_{0},\pi_{1},\ldots ,\pi_{d}\right)=0$$

where each $\pi_{i},\;i\;=\;0,\;1,\;\ldots ,\;d$ is a dimensionless product [56]. Buckingham’s π-theorem (Theorem 1) states that the physical phenomena will be now described by n−k = d number of dimensionless products.

**Theorem** **1.** *Buckingham’s π-theorem states that:*

*When a complete relationship between dimensional physical quantities is expressed in dimensionless form, the number of independent quantities that appear in it is reduced from the original n to n − k, where k is the maximum number of the original n that are dimensionally independent.*

In the dimensionless causal form of dimensional analysis [57]:

(4) $$\pi_{0}=f\left(\pi_{1},\pi_{2},\ldots ,\pi_{d-1}\right)$$

The dependent dimensionless product π_0_ will be a function of the set of independent dimensionless products $\pi_{1}{,\;\pi }_{2}{,\;\ldots ,\;\pi }_{d-1}$. The particular form of the function in dimensionless causal relationship is not provided by Buckingham’s π-theorem and should be determined experimentally [58]. The independent dimensionless product is divided in proper domains and a power law form is adopted to fit the results, as the following [57]:

(5) $$\pi_{0}=C\pi_{1}^{\alpha }\pi_{2}^{\beta }\cdots \pi_{d-1}^{\delta }$$

where *C* is a proportionality constant, and α, β,…, δ are real numbers. Both are defined for the working domain of independent dimensionless products.

#### 2.1.2. Selective Laser Melting Dimensional Analysis

The independent variables that have the most influence on the final density of parts produced via SLM, and thus are chosen as independent physical quantities for the dimensional analysis developed in this work, are volumetric energy density (γ), average particle diameter (ϕ), scanning speed (v), specific heat capacity ($C_{p}$), and heat conductivity (κ). VED represents the energy input for ensuring proper melt of the powder. If scanning speed is improperly high, adequate densification will not be achieved as proper melt may not occur. Furthermore, if scanning speed is too low, powder particles will be ejected from the powder bed. Heat conductivity is of the upmost importance as heat conduction is one of the governing phenomenon in selective laser melting. Specific heat capacity is highly relevant since it is related to the required energy to raise the material’s temperature.

In this work, VED (γ) is defined as:

(6) $$\gamma =\frac{P}{vht}$$

where *P* is the laser power (W), *v* is the scanning speed (m/s), *h* is the hatch spacing (m), and *t* is the layer thickness (m). Definition of fundamental dimensions along with symbol, units, and dimensions of factors are summarized in Table 3 and Table 4, respectively.

Volumetric energy density, scanning speed, average particle diameter, and specific heat capacity are chosen as the dimensionally independent subset. The equidimensional products of heat conductivity and density are defined in Equations (7) and (8): is introduced:

(7) $$\left[\kappa \right]=\;\left[\gamma \right]{\left[v\right]}^{-1}\left[C_{p}\right]\left[\phi \right]$$

(8) $$\left[\rho \right]=\left[\gamma \right]{\left[v\right]}^{-2}$$

Afterwards, the dependent and independent dimensionless products, presented in Equations (9) and (10), respectively, are set using the quotient of the remaining dependent and independent variables with their respective equidimensional product:

(9) $$\pi_{0}=\frac{\rho v^{2}}{\gamma }$$

(10) $$\pi_{1}=\frac{\kappa v}{\gamma C_{p}\phi }$$

Using Buckingham’s π-theorem, the following expression for determining the bulk density of metallic pieces produced by selective laser melting is obtained:

(11) $$\rho =C\left(\frac{\gamma }{v^{2}}\right){\left(\frac{\kappa v}{\gamma C_{p}\phi }\right)}^{\alpha }$$

where α and *C* are found by fitting experimental data. We shall discuss this process in the next section.

### 2.2. Experiments

**Figure 1:**
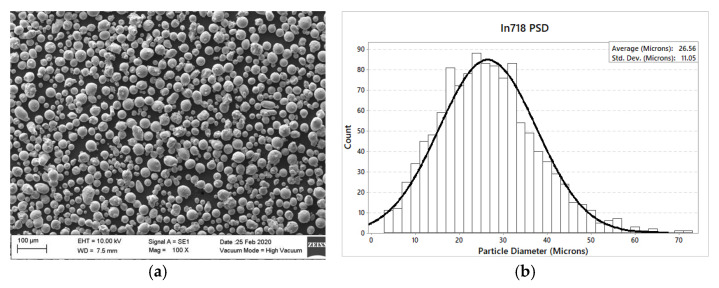
Praxair’s TruForm In718 powder’s (**a**) scanning electron microscopy image and (**b**) particle size distribution.

Truform In718 powder was acquired from Praxair (Truform 718 metal powder, Monterrey, Mexico). The overall chemical composition provided by the supplier is summarized in Table 5. The alloy is estimated to possess a hardness (Brinell, HB) value ranging from 240–410 kg/mm^2^. Figure 1 shows scanning electron microscopy (SEM) images of the powder as received. The average powder diameter is determined to be of 26.56 µm. Figure 1b illustrates the powder size distribution. Notice from Figure 1a that the powder morphology is of spherical-like shape. However, it is not uniform among the powder particles. Next, the Inconel 718 density, taken from Aerospace Specification of Metals, was fixed to the value of 8190 kg/m^3^. Specific heat capacity and thermal conductivity were estimated to have the values of 435 W/M·°K and 11.4 J/kg·°K, respectively.

**Figure 2:**
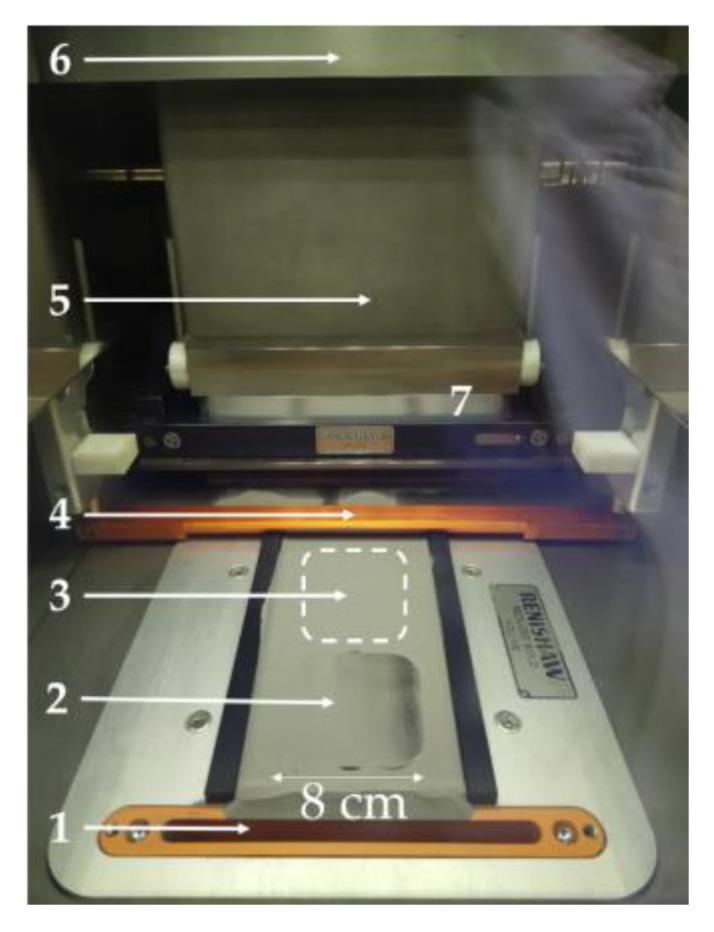
Experimental set-up for the selective laser melting of In718 cubes. The main elements of the experimental set-up are marked: (1) overflow, (2) build plate, (3) powder feed container, (4) recoater, (5) inert atmosphere; argon, (6) laser and optics system; Nd: YAG fiber laser, and (7) building chamber.

In718 cubes (10 mm × 10 mm × 10 mm) were manufactured using Renishaw AM400 system (Wharton, UK) equipped with a 400 W, pulsed wave, Nd:YAG fiber laser (wavelength of 1080 nm and laser focus diameter of 70 µm). Figure 2 depicts the experimental set-up, along with its main elements, where the selective laser melting of the probes was carried out. It is first ensured that the building chamber is in an inert atmosphere, achieving so, by flooding it with argon gas. A homogeneous layer of metallic powder (Inconel 718) is first spread, by the recoater (Reduced Build Volume equipment, Renishaw, Wharton, UK), on the build plate. The excess powder is dragged by the recoater to the overflow, where it is stored for further potential reuse. A laser beam, as heat source, selectively scans the build plate, melting a cross section of the final product. After solidification, the build plate lowers, the recoater returns to its initial position, and the powder feed container rises. The process is repeated until the final piece is constructed.

Layer thickness (*t*), hatch spacing (*h*), and point distance (*p_d_*) were set to 60, 70, and 70 µm, respectively. Laser power (*P*) and exposure time (*t_on_*) were varied from 360 to 400 W and 35 to 40 µs, respectively. Scanning speed (*v*) was approximated, referring to the work of Tiwari et al. [14], as the quotient between point distance and exposure time for compatibility with a continuous wave laser. A summary of the parameters used, along with VED (*γ*), are listed in Table 6. In total, 27 probes were manufactured: 3 probes per experimental condition. The results presented are the mean average values recorded from 3 specimens of the same batch. After fabrication, bulk density of the built specimens was measured with Mettler Toledo XPR Analytical Balance (Zurich, Switzerland) equipped with its density measuring kit through buoyancy method.

## 3. Results and Discussion

**Figure 3:**
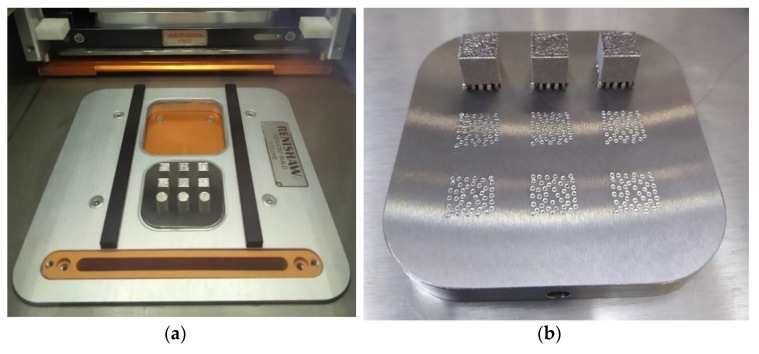
SLMed Inconel 718 cubes in (**a**) the build chamber after construction and (**b**) build plate on the fabrication of another set of experimental probes. Good surface finish is observed.

Manufactured samples, in the building chamber and in the build plate of a different set of experimental probes, are shown in Figure 3a,b, respectively. After fabrication, the samples were manually removed from the build plate.

Bulk density experimental measurements of the fabricated specimens are listed in Table 7 along with the relative density values measured. The maximum relative density value of 96.082% in specimen ID 33 was obtained under the following machine process parameters: laser power of 400 W, 40 µs of exposure time (scanning speed of 1.75 m/s), and a volumetric energy density of 54.42 × 10^9^ J/m^3^. The specimen with lowest relative density (94.274%) was produced considering the following process parameter values: laser power of 400 W, 35 µs of exposure time (a scanning speed of 2 m/s), with a volumetric energy density of 47.62 × 10^9^ J/m^3^. In average, a densification of 95.218% was experimentally measured.

**Figure 4:**
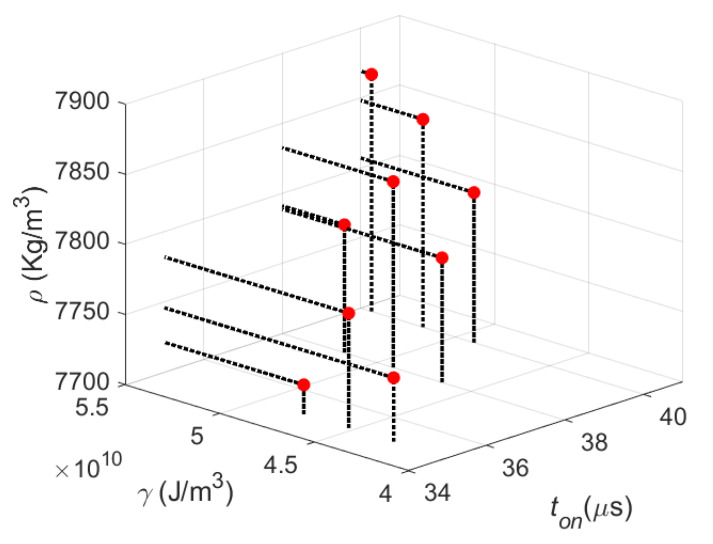
Graph of the resulting bulk density (ρ) with respect to exposure time (*t_on_*) and volumetric energy density (γ). In general, it is observed that higher bulk density values are attained at higher exposure time and volumetric energy density conditions. Please notice that the volumetric energy density (γ) values, in J/m^3^, are in the ×10^10^ range.

Figure 4 shows a graph of the resulting bulk density (ρ) with respect to exposure time (*t_on_*) and volumetric energy density (γ). It is observed, in overall, that with higher exposure time values, better densification is achieved. If the laser remains in contact with the metallic material for longer periods, it is easier for the heat addition to be sufficient to properly melt the powder. In general, higher bulk density values in SLM are obtained at higher conditions of volumetric energy density. The VED concept provides information of the energetic input needed to ensure full melt of the metallic powder, while avoiding the sublimation and ejection of powder particles.

As previously stated, scanning speed (*v*) is defined as the quotient between point distance (*p_d_*) and exposure time (*t_on_*). The following, Equation (12):

(12) $$\rho =C\left(\frac{\gamma {t_{on}}^{2}}{p_{d}^{2}}\right){\left(\frac{\kappa p_{d}}{\gamma C_{p}\phi \;t_{on}}\right)}^{\alpha }$$

is obtained when substituting this definition into Equation (11). Equation (12) is useful for visualizing the effect of exposure time and point distance in the bulk density of components produced by selective laser melting. Equation (12) differs from Equation (11) in that, the latter one is more adequate for SLM where a continuous wave laser is used. On the other hand, Equation (12) is compatible with a pulsed wave laser SLM machine. From Equation (12), it may be concluded that exposure time, as well as volumetric energy density, will have a positive influence on bulk density, as observed simultaneously in Figure 4. This conclusion is equivalent to arguing that working at lower scanning speed conditions will produce components of higher density. The only difference is that scanning speed is used for a continuous wave laser, and the conjunction of point distance and exposure time is used for pulsed wave lasers.

**Figure 5:**
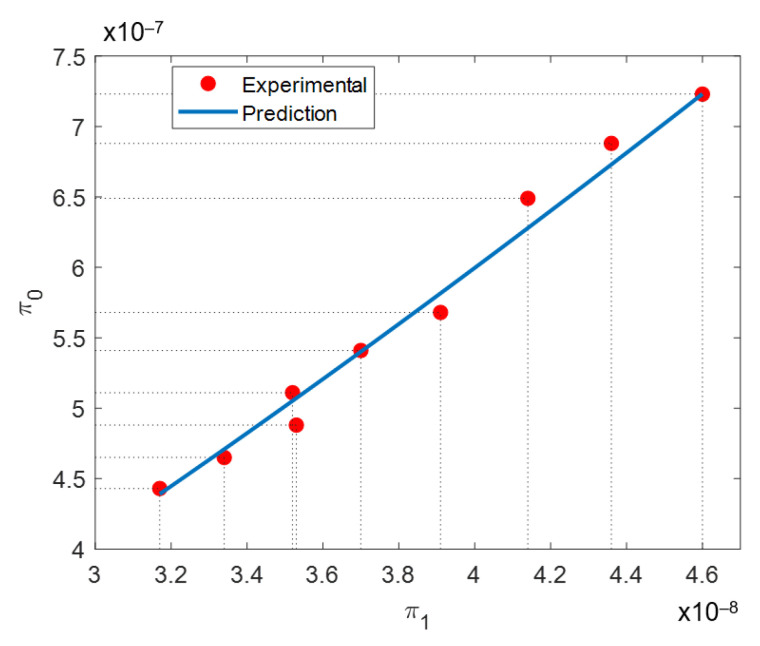
Experimental plot of π_0_ (dependent) vs. π_1_ (independent) dimensionless numbers. The correlation between π_0_ and π_1_ is an indication that dimensional analysis has succeeded in describing the physics of the SLM process [57].

Substituting the values of *γ*, ρ, and *v* listed in Table 6 and Table 7 and the material thermal conductivity value of *κ* = 11.4 J/kg·°K, and its specific heat capacity *C_p_* = 435 W/m·°K, alongside with the measured average particle diameter ϕ = 26.56 μm into Equations (9) and (10), yields the experimental points shown in Figure 5. Then, the experimental data were fitted following the classical nonlinear least square method from which the values *C* = 4910.4 and α = 1.34 were found for the working independent dimensionless range of π_1_ between 3.17 × 10^−8^ to 4.6 × 10^−8^. Thus, the SLMed In718 samples’ bulk density can be predicted using the following equation:

(13) $$\rho =4910.4\left(\frac{\gamma }{v^{2}}\right){\left(\frac{\kappa v}{\gamma C_{p}\phi }\right)}^{1.34}$$

Equations (9) and (10) can be reduced to the simple dimensionless relationship:

(14) $$\pi_{0}=4910.4{\pi_{1}}^{1.34}$$

From Equation (13), it is observed that dimensional analysis has provided an insight on the attained bulk density for the SLM process. In this case, the bulk density is positively affected by VED and inversely by scanning velocity. The independent dimensionless product π_1_ is found to influence bulk density with a power of 1.34. Based on these results, it is now evident from Equation (13) how the bulk density varies as a function of the velocity, heat conductivity, VED, specific heat capacity, and powder particle diameter. It is important to highlight that dimensional analysis states that it must be considered that the dimensionless form of bulk density, in Equation (9), is affected by π_1_ as a whole and not by its individual elements alone [57].

Following Buckingham’s π-theorem, the process initially described by six factors is now described by two i.e., a dependent (π_0_) and an independent (π_1_) dimensionless product. Dimensionless density product π_0_ incorporates density, scanning speed, and VED. The two latter are closely related to the energy input received by the powder bed. Independent dimensionless product π_1_ includes energetic input factors such as scanning speed and VED, powder thermal properties such as thermal conductivity and specific heat capacity, and metallic powder particle average diameter value.

A curve of the dependent π_0_ and independent π_1_ dimensionless products along with the prediction provided by Equation (14) is illustrated in Figure 5. From Figure 5, a strong correlation between π_0_ and π_1_ is observed. This is a proof that dimensional analysis has succeeded in describing the physic of the SLM process with dimensionless products. Equation (9) depicts the expression for the dependent dimensionless product, π_0_. The value it attains will be a function of resulting bulk density, the laser scanning speed, and the volumetric energy density conditions. Greater values of bulk density and laser scanning speed will produce a larger π_0_ value. Volumetric energy density has the opposite effect. For π_1_, presented in Equation (10), thermal conductivity (*κ*), specific heat capacity (*C_p_*), and average grain diameter (ϕ), remain unchanged in all experimental points. Then, the only parameters affecting π_1_ are laser scanning speed and volumetric energy density. Again, greater laser scanning speed will produce greater π_1_ values, while the opposite will happen with greater volumetric energy density. Laser scanning speed will affect both dependent and independent dimensionless products. However, it will have a greater influence on π_0_ than in π_1_.

**Figure 6:**
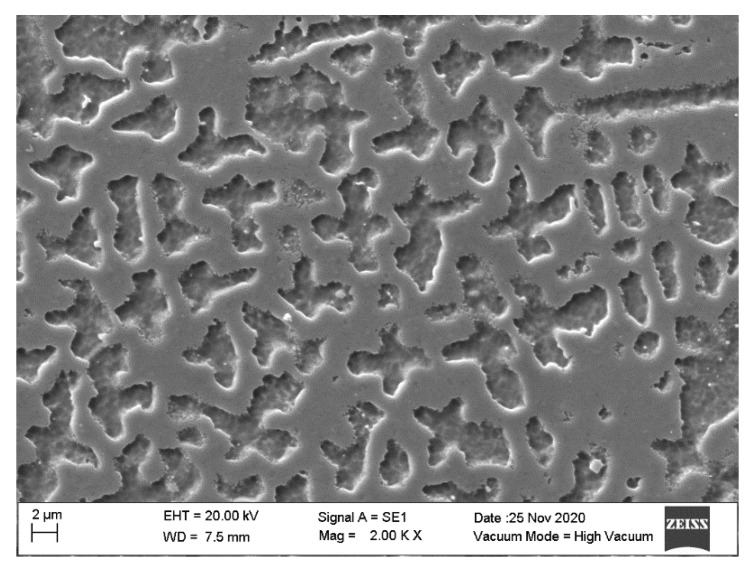
SEM image of the top surface of the In718 manufactured probe with ID 33.

Figure 6 presents a SEM image of the top surface of the In718 manufactured probe with ID 33, for which experimental conditions are described in Table 6. Evaluating Equation (13) with the manufacturing process parameters and physical properties, the developed mathematical model yields that the resulting bulk density will be of ρ = 7811.9724 kg/m^3^. Nevertheless, as shown in Table 7, the real value of bulk density is 7869.09 kg/m^3^. An error percentage between experimentally measured bulk density and the prediction provided by the developed mathematical model is of 0.7259%.

Experimental bulk density and dependent dimensionless product, along with theoretical prediction obtained from Equation (13), and the corresponding error percentages are listed in Table 8. Here, an average percentage error of 1.6503% is attained. The biggest errors of 3.7119% and 3.0542% were observed in specimens 13 and 31. However, high precision was achieved with a maximum error values of 0.1611% and 0.1617% in specimens 22 and 11, respectively. Therefore, it is concluded that the expressions derived from dimensional analysis fit experimental data well.

**Figure 7:**
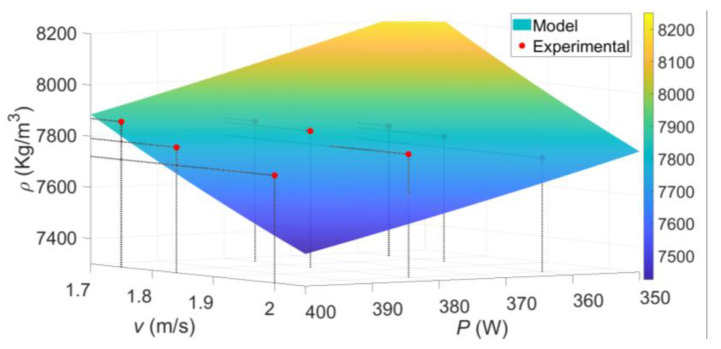
Experimental and predicted samples’ density graph as a function of laser power and scan velocity. It is observed that the best densification conditions are obtained at lower scanning speeds and small laser power. Low scanning speed implies larger exposure time of the laser in the powder bed which increases sample densification. When the laser power supply is improperly set, it creates undesirable effects such as particle sublimation or ejection, which leads to metallic samples with higher porosity.

A surface plot of bulk density with respect to laser power and scanning velocity is shown in Figure 7, where it is evident that bulk density increases with lower scanning speed and tends to decrease with increasing laser power. Higher scanning speed implies less exposure time of the laser to the powder bed which is related to energy input and sample densification. High laser power values may be related to powder particle sublimation and ejection affecting densification [52]. It is important to point out that the specific values of *C* and α are only valid in the range interval values of *π*_1_ from 3.17 × 10^−8^ to 4.6 × 10^−8^, determined according to the manufacturing parameter values used during the fabrication of the metallic samples.

An equation to estimate scanning speed with respect to laser power supply to obtain the desire sample densification is obtained by substituting Equation (6) into Equation (13), and using theoretical density value (ρ_TH_) instead of bulk density:

(15) $$v={\left(P^{1-\alpha }\frac{C}{\rho_{\mathrm{TH}}ht}{\left(\frac{\kappa ht}{C_{p}\phi }\right)}^{\alpha }\right)}^{\frac{1}{3-2\alpha }}$$

Equation (15) is valid for any independent dimensionless product range value of *π*_1_ because of the dimensional analysis. Thus, solving Equation (15) for *P*, yields:

(16) $$P={\left(v^{2\alpha -3}\frac{C}{\rho_{\mathrm{TH}}ht}{\left(\frac{\kappa ht}{C_{p}\phi }\right)}^{\alpha }\right)}^{\frac{1}{\alpha -1}}$$

**Figure 8:**
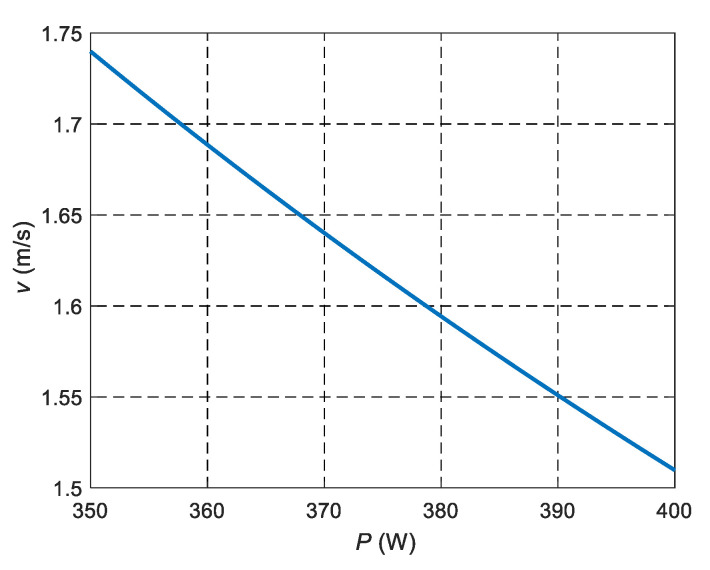
Scanning speed with respect to laser power supply needed to achieve high densification of SLMed Inconel 718 samples. This plot is useful for tuning scanning velocity with respect to laser power supply for the SLM of Inconel 718 in an independent dimensionless product range π_1_ from 3.17 × 10^−8^ to 4.6 × 10^−8^.

In this case, Equation (16) can be used to predict the laser power supply as a function of the scanning speed needed to achieve high densification on the AM metallic sample, as shown in Figure 7 for Inconel 718. Specific recommendations for the definition of laser scanning velocity, with respect to laser power, to SLM In718 pieces may be drawn from Figure 8. For example, with the specific conditions described in the experimental set up of this work, if a laser power of *P* = 390 W is defined, a laser scanning velocity of *v* = 1.55 m/s is needed to achieve highly dense components. Likewise, if a laser scanning velocity of *v* = 1.7 m/s is defined, an approximate laser power value of *P* ≈ 357 W will do so too.

To further validate our proposed model, let us consider experimental data collected by Kempen et al. [59] during the AM of metallic samples made from AlSi10Mg alloy, of chemical composition provided in Table 9. For this material, the following parameter values were assumed: *κ* = 110 W/m·°K, *C_p_* = 910 J/kg·°K, ρ_TH_ = 2680 kg/m^3^, with particle average diameter value of 16.3 μm. Manufacturing parameters used in the calculation of the dimensionless products are listed in Table 10. Therefore, Equation (16) can be used to determine the laser power supply needed to manufacture highly dense AlSi10Mg metallic samples as a function of the scanning speed.

**Figure 9:**
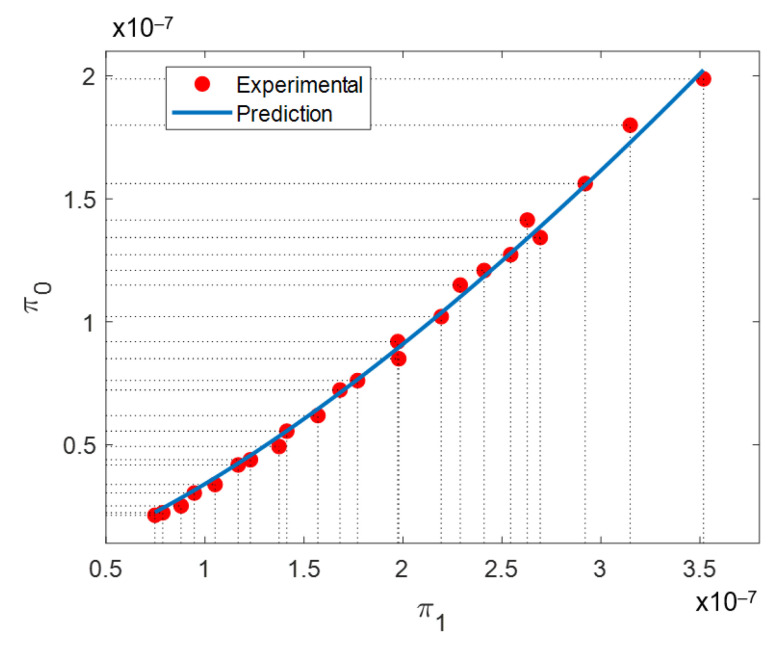
Plot of π_0_ vs. π_1_ using experimental data collected by Kempen et al. in [59] during the fabrication of metallic samples made from AlSi10Mg alloy.

The values of *C* = 278.4 and α = 1.416 with an RMSE of 3.7023 × 10^−9^ were calculated by fitting experimental data using a nonlinear least square method. Figure 9 shows the curve obtained using the dependent and independent dimensionless products. Notice that the proposed dimensionless mathematical model captures well the SLM process of AlSi10Mg alloy samples, too.

**Figure 10:**
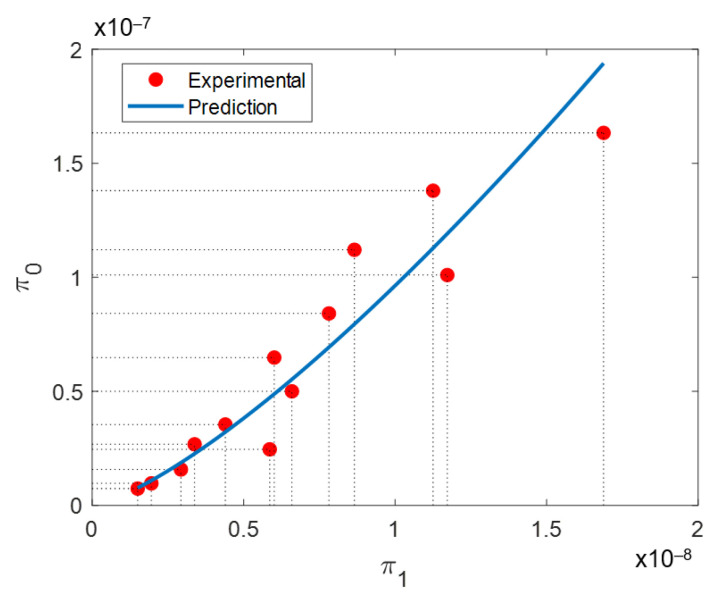
Plot of π_0_ (dependent) vs. π_1_ (independent) dimensionless numbers calculated using experimental data collected during the fabrication of Ti6Al4V metallic via SLM [60].

Similar conclusions can be drawn for Ti6Al4V samples produced with the SLM process, as shown in Figure 10. In this case, the material parameter values are: *κ* = 6.4 W/m °K, *C_p_* = 546 J/kg °K, ρ_TH_ = 4220 kg/m^3^, with ϕ *=* 30 μm [60]. The chemical composition of Ti6Al4V powder, reported by the provider in [61], used by Dilip et al. is presented in Table 11. It is easy to show that the values of *C* and α are, using the parameter values listed in Table 12, 4612 and 1.335, respectively, with a RMSE value of 2.0842 × 10^−9^.

## 4. Conclusions

In this article, a general expression for determining the scanning speed needed to achieve the part’s high densification as a function laser power supply was derived using Buckingham’s π-theorem dimensional analysis. The derived expression allows to identify how powder material properties and SLM process parameters are connected via the interaction between the dimensionless groups π_0_ and π_1_.

The accuracy of the derived expression that relates π_0_ and π_1_ is assessed considering additive manufactured In718 samples. Collected experimental data were used to plot π_0_ vs. π_1_ finding a correlation between them, which is an indication of the validity of Equation (13). In fact, the error percentage value attained between experimental data and predicted bulk density values does not exceed 3.71%.

The effect of relevant manufacturing parameters in SLM was assessed. It was found that greater exposure time, of the laser beam on the powder bed (or lower laser scanning speed), leads to better densification. It was also shown that volumetric energy density has an overall positive influence on bulk density. The negative effect of laser power in bulk density is attributed to improperly high energetic conditions which cause the sublimation and ejection of powder particles for the specific experimental manufacturing parameters framework.

Moreover, using the expression that defines the volumetric energy density and the expressions that define π_0_ and π_1_, we were able to find a relationship between the scanning speed and the laser power supply. This expression sheds a new light on how AM process parameters and powder material properties are connected, since the applicability of the derived expression through dimensionless analysis could help the user in tuning machine process parameters in such a way that the end part could attain the desirable bulk density.

Finally, the developed mathematical model based on Buckingham-π theorem is able to properly predict collected data obtained during the additive manufacturing via SLM of In718, AlSi10Mg, and Ti6Al4V metallic samples. Therefore, this paper provides evidence of the applicability of the proposed dimensionless model for AM metallic parts produced by SLM in industrial sectors such as aerospace, medical devices, industrial design, and automotive, to name a few.

In comparison with previous attempts of applying dimensional analysis to describe the physical process of selective laser melting, this work has developed expressions of high applicability and practical relevance. The chosen set of independent physical quantities is significantly smaller than the previously proposed ones. This has allowed the full evaluation of the physical model with an experimental stage. Highly precise predictions on the bulk density of SLMed components, with respect to relevant process parameters and powder properties, have been able to be drawn from the developed mathematical expressions. Moreover, it is now possible to identify the scanning speed value needed to achieve high part densification, with respect to the laser power supply, leading to a potential significant reduction on material waste and costs associated to experimentation.

## Figures and Tables

**Table 1:** Experimental investigations on the effect of relevant process parameters in SLM.

SLM Process Parameter	Studied Property	Material	Reference
Laser power	Porosity and hardness	AlSi10Mg/graphene	[18]
	Grain orientation	CoCrMo	[19]
	Porosity	AlSi10Mg	[20]
	Densification	Ti64	[21]
Scanning speed	Densification	SS316L	[22]
	Relative density, melt pool depth, and hardness	Maraging steel	[23]
	Porosity	AlSi10Mg	[20]
Scan spacing	Densification	SS316L	[22]
	Relative density, melt pool depth, and hardness	Maraging steel	[23]
	Porosity	AlSi10Mg	[20]
	Densification	Ti64	[21]
PSD	Mechanical properties and microstructure	Inconel 625	[24]
	Powder to laser absorptivity	W	[25]
Powder flowability	Mechanical properties and microstructure	Inconel 625	[24]
Powder morphology	Hardness and microstructure	TiB2/SS316L	[26]
Layer thickness	Mechanical, fractographic, and compositional properties	CoCr	[27]
Exposure time	Densification	Ti64	[21]
Point distance	Densification	Ti64	[21]

**Table 2:** Studies on the effect of volumetric energy density in SLM.

SLM Process Parameter	Studied Property	Material	Reference
Volumetric Energy Density	Melt pool dimensions and geometry	Ti6Al4V	[30]
	Densification	W	[31]
	Density, surface roughness, and dimensional accuracy	AlSi10Mg and Al6061	[32]
	Microstructure, porosity, and microhardness	Inconel 718	[33]
	Densification and fatigue life	Inconel 625	[34]
	Densification and hardness	SS316L	[35]
	Porosity	Cu	[36]
	Melt pool shape	300M steel	[37]
	Tensile properties	Ti6Al4V	[38]
	Texture anisotropy and mechanical properties	Inconel 718	[39]
	Microstructural evolution, texture, and mechanical properties	SS304L	[40]

**Table 3:** Symbol and unit of the fundamental dimensions involved in the dimensional analysis.

Dimension	Symbol	Unit
Time	T	s
Length	L	m
Mass	M	kg
Temperature	θ	°K

**Table 4:** Units, symbol, and dimensions of the physical quantities involved in the dimensional analysis of selective laser melting.

Factor	Symbol	Units	Dimensions
Volumetric energy density	γ	J/m^3^	*ML* ^−1^ *T* ^−2^
Average particle diameter	ϕ	m	*L*
Scanning speed	*v*	m/s	*LT* ^−1^
Specific heat capacity	*C_p_*	J/kg·°K	*L*^2^*T*^−2^θ^−1^
Heat conductivity	*κ*	W/m·°K	*MLT*^−3^θ^−1^
Bulk density	ρ	kg/m^3^	*ML* ^−3^

**Table 5:** Truform Inconel 718 metal powder chemical composition.

Element	Ni	Cr	Fe	Nb + Ta	Mo	Ti	Al	Co	Mn	Si	Cu
Composition (*Max)	50–55	17–21	15–21	4.7–5.5	2.8–3.3	0.6–1.1	0.2–0.8	1 *	0.35 *	0.35 *	0.3 *

* Implies maximum percentage of chemical composition.

**Table 6:** Fabrication parameter values.

ID	*P* (W)	*t_on_* (µs)	*v* (m/s)	γ (J/m^3^) (1 × 10^9^)
11	360	35	2	42.86
12	360	38	1.84	46.53
13	360	40	1.75	48.98
21	380	35	2	45.24
22	380	38	1.84	49.11
23	380	40	1.75	51.70
31	400	35	2	47.62
32	400	38	1.84	51.70
33	400	40	1.75	54.42

**Table 7:** Bulk and relative density of the fabricated specimens.

ID	Bulk Density (kg/m^3^)	Relative Density
11	7745.66	0.94575
12	7788.83	0.95102
13	7807.09	0.95325
21	7781.87	0.95017
22	7832.60	0.95636
23	7848.19	0.95826
31	7721.01	0.94274
32	7791.15	0.95130
33	7869.09	0.96082

**Table 8:** Comparison between experimental and predicted values for ρ and π_0_. Notice that the maximum error attained does not exceed 3.71%, which is an indication of the accuracy of the proposed dimensionless model.

ID	π_1_ (×10^–8^)	Exp. π_0_ (×10^–7^)	Mod. π_0_ (×10^–7^)	Exp. ρ (kg/m^3^)	Mod. ρ (kg/m^3^)	Error ptg.
11	4.60	7.23	7.24	7745.6633	7758.1974	0.1617%
12	3.91	5.68	5.81	7788.8333	7965.0737	2.2626%
13	3.53	4.88	5.06	7807.0933	8096.8901	3.7119%
21	4.36	6.88	6.73	7781.8700	7616.8824	2.1202%
22	3.70	5.41	5.40	7832.6033	7819.9905	0.1611%
23	3.34	4.65	4.71	7848.1867	7949.4059	1.2896%
31	4.14	6.49	6.29	7721.0067	7485.1977	3.0542%
32	3.52	5.11	5.04	7791.1500	7684.7944	1.3652%
33	3.17	4.43	4.40	7869.0900	7811.9724	0.7259%

**Table 9:** Chemical composition of AlSi10Mg powder used in [59].

Element	Al	Si	Fe	Cu	Mg
Composition	90.38	9.02	0.123	0.006	0.471

**Table 10:** Parameter values used to AM AlSi10Mg alloy samples. Experimental data taken from [59].

ID	*v* (m/s)	γ (J/m^3^) (1 × 10^9^)	Relative Density	Bulk Density (kg/m^3^)
1	0.8	75.40	0.9835	2635.7264
2	1	60.32	0.9887	2649.5820
3	1.2	50.26	0.9915	2657.0860
4	1.4	43.08	0.9913	2656.6036
5	1.6	37.70	0.9887	2649.7964
6	0.9	63.49	0.9902	2653.8432
7	1.1	51.95	0.9915	2657.3272
8	1.3	43.96	0.9910	2655.7460
9	1.4	40.82	0.9891	2650.8148
10	1.5	38.10	0.9868	2644.5436
11	0.8	67.46	0.9907	2655.0492
12	1	53.97	0.9931	2661.5080
13	1.2	44.97	0.9907	2655.0224
14	1.4	38.55	0.9853	2640.6040
15	1.6	33.73	0.9772	2618.7888
16	0.8	79.37	0.9883	2648.5368
17	0.9	70.55	0.9882	2648.4028
18	1	63.49	0.9905	2654.4328
19	1.1	57.72	0.9887	2649.7964
20	1.2	52.91	0.9911	2656.1480
21	1.3	48.84	0.9916	2657.4612
22	1.4	45.35	0.9925	2659.8464
23	1.5	42.33	0.9924	2659.6320

**Table 11:** Chemical composition, reported by the supplier [61], of Ti6Al4V powder used in [60].

Element	Ti	Al	V	Fe	O	C	N	H
Composition	0.88–0.9	5.5–6.5	3.5–4.5	<0.25	<0.13	<0.08	<0.05	<0.012

**Table 12:** Parameter values used to AM Ti6Al4V alloy samples. Experimental data taken from [60].

ID	*v* (m/s)	γ (J/m^3^) (1 × 10^9^)	Relative Density	Bulk Density (kg/m^3^)
1	0.5	33.33	0.7764	3276.5346
2	0.5	66.67	0.9959	4202.4870
3	0.5	100.00	0.9160	3865.3090
4	0.5	130.00	0.9074	3829.3124
5	0.75	44.44	0.9362	3950.9328
6	0.75	66.67	0.9959	4202.4870
7	0.75	86.67	0.9778	4126.4426
8	1	33.33	0.7976	3365.9986
9	1	50.00	0.9970	4207.1290
10	1	65.00	0.9982	4212.4040
11	1.2	27.78	0.7465	3150.3144
12	1.2	41.67	0.9459	3991.8246
13	1.2	54.17	0.9989	4215.3158

## Data Availability

Data available on request due to restrictions eg privacy. The data presented in this study are available on request from the corresponding author.

## Nomenclature

Acronyms/Variables	Definition
SLM	Selective Laser Melting
SLS	Selective Laser Sintering
AM	Additive Manufacture
3D	Three-dimensional
SEM	Scanning Electron Microscopy
FEM	Finite Element Method
RMSE	Root Mean Square Error
PSD	Particle Size Distribution
*γ*/VED	Volumetric energy density, J/m^3^
*v*	Scanning speed, m/s
*P*	Laser power, W
*κ*	Heat conductivity, J/m·°K
*C_p_*	Specific heat capacity, J/kg·°K
ϕ	Average powder diameter, m
ρ	Bulk density, kg/m^3^
ρ_TH_	Theoretical density, kg/m^3^
*t_on_*	Exposure time, s
*h*	Hatch spacing, m
*t*	Layer thickness, m
*p_d_*	Point distance, m
π_0_	Dependent dimensionless product
π_1_	Independent dimensionless product
*C*	Proportionality constant
α	Fitting exponent
